# Supporting sexual minority adolescents: A critical realist thematic analysis of psychological therapists' experiences

**DOI:** 10.1111/papt.70051

**Published:** 2026-03-05

**Authors:** Paris Young, Nargis Islam, James Lea, Anna Daiches

**Affiliations:** ^1^ Lancaster University Lancaster UK; ^2^ University of Manchester Manchester UK

**Keywords:** clinical psychology, critical realism, psychological therapists, psychotherapy, qualitative research, sexual minority adolescents

## Abstract

**Objectives:**

Sexual minority adolescents experience elevated rates of psychological distress, influenced by societal stigma and heteronormativity. Psychological therapists can play a key role in supporting identity development and advocating for systemic change. This study explored therapists' lived experiences of working with sexual minority adolescents, attending to both reported experiences and deeper social mechanisms.

**Design:**

A qualitative study using a critical realist approach to thematic analysis that integrated inductive coding with abductive and retroductive theorising.

**Methods:**

Seven UK‐based psychological therapists participated in semi‐structured interviews conducted between January and March 2024. Analysis examined both dispositional and inferential themes, exploring both surface experiences and deeper social mechanisms shaping participants' experiences.

**Results:**

Three dispositional themes were generated: (1) therapists' experiences of socio‐environmental forces shaping adolescent sexuality; (2) the striving to offer attuned, responsive therapy; and (3) the influence of socio‐political tensions on navigating identity‐related work. The analysis suggests that the therapeutic process functions as a *relational shield* against conflicting structural forces. By centring this relational process to navigate developmental fluidity, effective practice relied on *agential striving* to protect the adolescent's narrative from external agendas. This involved fundamental clinical skills: creating a non‐judgemental space, using compassionate curiosity, and maintaining awareness of relational pulls and assumptions.

**Conclusions:**

Socio‐political structures are often enacted within the therapy room, presenting both challenges and opportunities for therapeutic work. The findings suggest that therapy functions as a mediating space, shielding adolescents' developing identities from polarisation and stigma. This highlights the ethical necessity of maintaining a protected process in a politicised climate.

## INTRODUCTION

Adolescence is a formative period in sexuality development, a process involving multiple milestones such as awareness of attractions, questioning and self‐identification (Hall, Dawes, & Plocek, 2021). For sexual minority adolescents (SMA), this journey is compounded by heightened social vulnerabilities like dependence on caregivers, peer rejection and limited supportive resources (Hall, Dawes, & Plocek, 2021; Russell & Fish, 2019). While many navigate this in isolation, peer connection can offer critical affirmation and positive outcomes (Kelleher, 2009; Scroggs & Vennum, 2021).

### Evolving social contexts

Following the declassification of same‐sex attraction as a mental disorder and the professional rejection of conversion therapy (APA, 2021; BACP, 2022; Barker, 2023; BPS, 2024; Graham, 2019), acceptance of sexual minorities has grown in many, particularly in high and middle‐income, countries (Bhugra et al., 2022). Growing media representation and digital platforms have enabled visibility and peer support (Bond, 2014; Pellicane et al., 2021), and younger generations are increasingly reporting as sexual minorities (ONS, 2023), a trend which may reflect either a demographic shift or a greater willingness to disclose previously concealed identities within a more accepting social climate.

Despite this progress, challenges remain. Media often reinforce stereotypes (Żerebecki et al., 2021), and SMA continue to be shaped by ‘heteronormativity’, a cultural framework positioning heterosexuality as the default or ‘natural’ norm (Robinson, 2016). Beyond the stigma that persists across many communities (Chiang et al., 2019; Heiden‐Rootes et al., 2021; Lin et al., 2020; Russell & Fish, 2019), same‐sex relationships remain criminalised in over 60 countries (Bandera, 2023). Notwithstanding legal protections in the United Kingdom, these contexts can profoundly affect self‐acceptance among SMA, especially those with direct or familial ties to such contexts.

### Health disparities

While minority sexual orientation is not inherently linked to poor health, SMA consistently report elevated mental health difficulties from early adolescence compared to heterosexual peers (Amos et al., 2020; Gilbey et al., 2020; Irish et al., 2019). Large population studies highlight increased psychological distress, self‐harm and suicide attempts, even when adjusting for socioeconomic factors (Khanolkar et al., 2023; Spittlehouse et al., 2020). Drawing on Brooks' (1981) foundational work concerning minority stress and lesbian women's experiences, which has been noted for receiving a ‘relative scarcity of scholarly attention’ (Rich et al., 2020, p. 124), Meyer's (2003) minority stress theory attributes these disparities to chronic stressors like rejection, bullying, and, for fear of these consequences, concealment (Ayhan et al., 2020; Baams et al., 2020; Kahle, 2020; Pachankis et al., 2020). This theory may explain some barriers to health care as reported by young people, including ‘stigma’, ‘don't want parent(s) to know’ and ‘previous experiences’ (Higgins et al., 2021).

Nevertheless, the theory has faced scrutiny. Diamond and Alley (2022), for example, critiqued its perceived deficit‐oriented perspective and limited evolutionary evidence base. They proposed *insufficient social safety* as a fuller explanation, where stigma acts as a primal threat that triggers self‐protective processes which harm long‐term health. Recently, Frost and Meyer (2023) engaged with this, positioning the social safety framework not as a replacement but as an augmentation of the original model. While acknowledging critiques of a deficit‐based focus, they also reiterated Meyer's (2014) earlier caution that an over‐emphasis on individual resilience can obscure the vital need for the ‘institutional investments required to support individuals’ (Frost & Meyer, 2023; Meyer, 2014, p. 349).

### Psychological professionals

Many have argued that interventions for sexual minorities are most effective if they begin in early adolescence (Amos et al., 2020; Gilbey et al., 2020; Irish et al., 2019). Psychological professionals, encompassing practitioner psychologists, psychotherapists and counsellors, play a central role in supporting SMA (Wampold & Imel, 2015). This role is guided within the UK by professional bodies such as the British Psychological Society (BPS) and the British Association for Counselling and Psychotherapy (BACP), which provide principles for working with sexual diversity (Barker, 2023; British Psychological Society [BPS], 2024). A key tenet is the importance of de‐centring sexuality, where therapists are reminded not to assume a client's difficulties stem from their identity (Barker, 2023), as most sexual minority people seek therapy for unrelated issues (Israel et al., 2008; Kelley, 2015). Therapists are encouraged to engage in self‐reflection (BPS, 2024) and acknowledge that distress often stems from marginalisation rather than identity itself (Barker, 2023; BPS, 2024). This allows them to support SMA in exploring sexuality‐related concerns and supporting positive identity development (Bruce et al., 2015; Jaspal et al., 2023; King, 2008).

Beyond individual therapy, this work extends to systemic interventions. This includes supporting social safety within the family, for instance by helping parents process barriers to accepting their children's sexuality and promoting understanding and closeness (Shpigel & Diamond, 2014). Furthermore, professionals are encouraged to work at an institutional level (BPS, 2024); those in influencing positions within health systems and education may raise awareness of the impacts of stigma and mobilise wider social change (Kinderman, 2014; Rog et al., 2021). However, existing UK guidance is explicitly scoped for adults (e.g. BPS, 2024), leaving a gap for psychological therapists working with the specific developmental complexity of SMA.

### Research question

Previous research on psychotherapeutic support for SMA has considered the effectiveness of interventions (e.g. Bochicchio et al., 2020), specific therapeutic approaches (e.g. Bafiti et al., 2019) and young people's perspective on facilitators and barriers to therapy (e.g. Heiden‐Rootes et al., 2021). From the therapists' perspective, research has largely focused on perceived gaps in training and competence (Carrington & Sims, 2024; Ho et al., 2023; King, 2008; Mathieson et al., 2023), leaving their broader clinical experiences and sense‐making processes largely unexplored.

This represents a significant academic and practical gap, which is further emphasised by the lack of specific UK‐based professional guidance for this adolescent population. Given that the therapist's subjective experience, clinical judgements and assumptions are instrumental in shaping the therapeutic alliance and process, understanding their perspective in‐depth is essential for informing effective professional development and supervision. This study asks: What is the experience of psychological therapists working with sexual minority adolescents? Specifically, it investigates what facilitates or hinders their work and how they navigate the therapeutic process within today's socio‐political landscape.

## METHODS

A qualitative approach was appropriate to address the research question to explore the experiences of psychological therapists. Data were collected via semi‐structured interviews and analysed using Wiltshire and Ronkainen's (2021) realist approach to thematic analysis, incorporating Braun and Clarke's (2006) approach. This study was approved by the Faculty of Health and Medicine Research Ethics Committee at Lancaster University (FHM‐2022‐0932‐RECR‐2).

### Philosophical underpinning

This study was informed by critical realism, which asserts that reality is intransitive and that it exists independent of the human knower (*ontological realism*), whereas knowledge is transitive and fallible since different interpretations of the same reality could be made depending on the perspective that it is viewed (*epistemological relativism*) (Bhaskar & Hartwig, 2016). Reality is viewed as stratified into the Empirical (experienced), the Actual (occurring whether observed or not), and the Real (causal mechanisms) domains (Fletcher, 2017; Wiltshire & Ronkainen, 2021). Causal mechanisms are understood as *tendencies* rather than fixed laws due to their context‐bound predictive power (Alderson, 2021; Bhaskar, 2008), underlining the interaction between social structures and human agency (Alderson, 2021).

In this study, the experiences of psychological therapists are observable and can be deductively captured (Empirical), whereas factors that may reside across the profession or in the social structures may be inferred from these experiences (Actual). Using *judgemental rationality* and thinking at higher levels of abstraction about causal mechanisms (Wiltshire & Ronkainen, 2021), factors that must exist in wider social structures for the participants' experiences to occur may be uncovered (Real).

### Participants

Participants were UK‐based, qualified and professionally registered, and had provided therapy in the past three years to at least one adolescent (13–19) reporting same‐sex attraction. There was no exclusion criteria based on participants' work contexts. Seven participants were recruited between December 2023 and March 2024 via email circulation within relevant services (NHS and independent services for young people), online professional networks, word‐of‐mouth and personal contacts. No compensation was offered to participants. Demographic characteristics are summarised in Table 1.

**TABLE 1:** Participant Characteristics (*n* = 7).

Pseudonym	Age	Gender	Profession	Years since qualifying	Sexuality	Ethnicity	Region	Clinical context(s)	Clinical area(s)
Alexis	43	Female	Clinical Psychologist	10	Heterosexual	White British	London	Independent	Children and adolescents
David	32	Male	Counselling Psychologist	6	Gay	White Scottish	Scotland	Independent, Education	Children and adolescents, Adults, Older adults, People who are neurodivergent
Jocelyn	41	Female	Clinical Psychologist	12	Heterosexual	White British	Northwest England	NHS, Independent, Education, Third sector	Children and adolescents, Working age adults
Moira	50	Other: ‘Gender is a social construction and not something I subscribe to’	Clinical Psychologist	23	Heterosexual	White British	Northwest England	Independent	Children and adolescents
Ron	30	‘Non‐binary’	CAMHS Practitioner	3	Gay	Mixed White and Asian	Northwest England	NHS	Children and adolescents, People who are neurodivergent
Stevie	56	Female	Clinical Psychologist	25	Heterosexual	White – Other: ‘*USA’*	Southeast England	Independent	Children and adolescents, Adults, People who are neurodivergent, Other: ‘*LGBTQ+ especially trans’*
Twyla	39	Female	Clinical Psychologist	5	Heterosexual	White British	Northwest England	Independent	Children and adolescents

To protect anonymity, the participants were each assigned a pseudonym. Only the lead author and designated administrative staff had access to identifiable information.

### Procedure

Interviews were conducted via Microsoft Teams between January and March 2024 using an interview guide focused on participants' experiences and the socio‐political context, consistent with a critical realist approach (Fryer, 2022) (see Supporting Information). Additional clarifying questions were asked based on their responses during the interviews.

After obtaining verbal consent, participants were reminded of confidentiality and withdrawal rights. With their verbal consent, the interviews were recorded and transcribed automatically by Microsoft Teams. The researcher then verified verbatim to correct errors or remove identifiable information. Each verified transcript was sent to the respective participant for member‐checking and, where appropriate, to suggest any removal of segments that might lead to their identity being identified or speculated.

### Data analysis

Analysis followed Braun and Clarke's (2006) six phases of thematic analysis, using an inductive and data‐driven approach (Braun & Clarke, 2022). Wiltshire and Ronkainen's (2021) critical realist framework guided the development of experiential, inferential and dispositional themes, corresponding to the Empirical, Actual and Real domains, respectively.

Initial experiential themes were generated in Nvivo 14. Themes were reviewed deductively across transcripts for recurrence, tracking regularities and strengths of each theme via a master spreadsheet. Inferential themes were then developed both inductively to identify narrative patterns and abductively to redescribe experiences abstractly (Fletcher, 2017; Wiltshire & Ronkainen, 2021). Dispositional themes were developed retroductively to theorise the causal social mechanisms shaping participants' experiences (Wiltshire & Ronkainen, 2021). Themes were refined iteratively in research meetings.

### Rigour

The lead author maintained a reflexive journal throughout data collection and analysis, discussed regularly in research meetings to identify potential biases. To enhance credibility, Wiltshire and Ronkainen (2021) suggested that experiential themes be adequately supported by the data and describe factual information closely representing the participants' experiences. Hence, experiential themes were returned to participants for validation and comments. Four responded with agreement and one suggested two changes to wordings, which were implemented. Higher‐order themes were examined for *ontological plausibility* and logical consistency (i.e. judgemental rationality).

### Reflexivity statement

While epistemological relativism suggests knowledge is contingent upon subjective interpretations (Bhaskar & Hartwig, 2016), Braun and Clarke (2022) considered researchers' subjectivity a valuable resource in the interpretation of meaning and active storytelling. As the lead author, I am a gay man and, at the time of the study, was a trainee clinical psychologist born in Hong Kong and educated in the United Kingdom. I have personal experience of navigating minority sexuality development in adolescence and have supported both SMA and adults clinically.

I approach socio‐political discourse with a cautious stance, alert to risks of polarisation and misinformation in the public sphere (see Barberá, 2020). As critical realism refutes the positivist assumption that research can be value‐free (Alderson, 2021), I recognise that suppressing political or moral values is impossible. Instead, I use reflexivity to critically manage these inherent biases, acknowledging that this is an ongoing process of learning and reflection to continuously challenge my own assumptions and professional blind spots.

## RESULTS

Three dispositional themes were developed to theorise the underlying mechanisms shaping psychological therapists' experiences of supporting SMA: (1) Psychological therapists' experience of the socio‐environmental forces shaping adolescent sexuality, (2) psychological therapists strive to offer attuned, responsive therapy and (3) wider socio‐political tensions influence how therapists navigate identity‐related work. Each dispositional theme is presented with its inferential themes and supported by experiential quotes (see Supporting Information).

### Psychological therapists' experience of the socio‐environmental forces shaping adolescent sexuality

This theme establishes the therapists' experience of encountering the powerful socio‐environmental structures that shape an adolescent's sexuality. Their accounts reveal that a primary part of their role involves witnessing the interplay between the young person's emerging agency and the often‐constraining forces, ranging from immediate peer dynamics to wider societal stigma.

#### Emerging sexual identity amid developmental complexity

Most participants viewed sexuality as ‘one part of a person, it's not all they are’ (Stevie) but acknowledged that it may be an important part. Most described it as emerging over time, with younger adolescents sometimes struggling to articulate their feelings. Older adolescents were sometimes described as more accepting of their sexuality, although this could be accompanied by more intense distress: ‘Where young people have been more distressed about [their sexuality]… there's been more shame built into how they're feeling… more often with older adolescents’ (Jocelyn).

#### Navigating stigma and social rejection

Participants consistently highlighted how immediate social context shaped identity development. While societal discourse has become more inclusive, they noted immediate environments like family and local community remained key to an adolescent's developing sense of self: ‘In this part of England where [being as a sexual minority] is even more accepted because it's so close to Brighton.… I was surprised that there was so much self‐hatred and shame’ (Stevie).

Some noted the impact of negative reactions from family members or hearing rejection directed at others: ‘[That shame] was due to another member of their family who had come out as gay and the way that they've been treated and talked about’. (Jocelyn) As a result, some participants cautioned against assuming that disclosure would always be helpful: ‘Sometimes that's true, and sometimes it isn't’. (Ron).

#### Adverse experiences shaping but not defining sexual identity

Some participants noticed an association between internalised stigma and adverse life experiences, such as bullying or rejection. Alexis reflected on supporting a young boy who experienced abuse by an older gay man, noting the complexity of formulation: ‘[He] potentially has internalised homophobia, but he also had this very extreme situation… that maybe plays into some homophobic stereotypes or tropes about gay men’.

Some believed SMA's ‘fear of judgement’ can be exaggerated by early rejections: ‘It was kind of wrapped up in how sensitive she was anyway, because of fear of rejection that had come with her from a really difficult background’ (Twyla), and peer rejection: ‘This early experience of liking another girl and that it went horribly wrong… put her off dating and made her really anxious’ (Alexis). However, these difficult experiences did not always lead to negative identity outcomes. Some SMA were able to draw clear boundaries between what had happened to them and how they saw themselves: ‘[They said,] “That happened to me but it's not who I am”.’ (Moira).

#### Fluidity and tensions in identity labels and self‐definition

Many participants described varied relationships with identity labels among SMA. For some, these labels supported self‐understanding and connection to others: ‘[They] like having a label to describe how they see themselves’, (Jocelyn) and a sense of belonging: ‘They feel like they're part of a community… of young people who identify as “queer”’. (Alexis) Others rejected fixed labels or preferred to remain undefined: ‘When I say, “That sounds like pan[sexuality]”, they go, “No, I'm just undefinable”.’ (Stevie) Many also reflected on how peer environments sometimes created pressure to identify a certain way, including discomfort with being heterosexual: ‘They often experience within their peer groups that there're particular answers that they should give in terms of their sexuality… that almost made it… uncool to be hetero[sexual]’. (Jocelyn).

A few participants acknowledged that language changes ‘all the time’ and ‘it's fine if you don't know what all of the words are’ (Ron). Many acknowledged that therapy should create space for fluidity and not impose expectations: ‘It's okay to just have those feelings and sit with them and see what happens. You don't have to decide [if] it's this label or that label’. (Ron).

### Psychological therapists strive to offer attuned, responsive therapy

Rather than a mere reaction to context, this theme explores how therapists' experience is shaped by a principled, dispositional striving to offer attuned, responsive therapeutic support. This agential striving involves a constant, active effort to manage their own internal worlds and relational pulls in order to create a protected therapeutic space that can withstand the external structural pressures faced by the young person.

#### Therapists' reflective inner dialogue

All participants spoke of not imposing their views or values, especially in work touching on identity: ‘You can offer something… but if your client doesn't take it, you don't push it because it's not on the client's agenda’ (David). This included not leading with their own politics, even when personally meaningful: ‘I'm a political person but my politics shouldn't be infiltrating my therapy space. When our ideologies come into the room, we don't leave space for that person's story’ (Moira). Similarly, Jocelyn said, ‘I'm not here to give [them] a perspective.… I'm here to [be] alongside them and help them make sense of their experience.’

Several recognised an internal pull to rescue but acknowledged that staying with uncertainty was crucial: ‘Jumping in rescuing would have helped [me] feel better, but it wouldn't have particularly solved some of the [young person's] challenges’. (Jocelyn) David, who identified as a gay man, shared an experience of managing defensiveness when a young person expressed internalised homophobia and said, ‘The thought of two gay men together makes me feel sick’. Through his use of curiosity and openness, the young person gradually developed a more nuanced perspective:

[The young person] ended therapy in a place where he wasn't, ‘Okay, I'm a gay man…’, but ‘I've done enough work on this for now. It's been really helpful to open my eyes to think about my sexuality from a less black‐and‐white place. I'm gonna have to wait and figure it out’. That felt like such a triumph. (David)

Twyla and Ron expressed disappointment when therapy ended before identity exploration could begin:

We'd come to the end… but he wasn't in a place where he thought he could even think about [being a sexual minority]…. I feel there was still somewhere that we needed to go, and I've let [him] down by not getting us to that point. (Ron).

Some said supervision was valued for helping them navigate these feelings: ‘He's still really young. [Ending therapy] doesn't mean he's never going to be in a place where he's ready to explore…. My supervisor is really good at telling me “You have to let go sometimes”’. (Ron).

#### Empowering adolescents in therapy

All participants consistently emphasised the need to keep SMA in the ‘driver's seat’ of the therapeutic process:

The client doesn't identify yet with this language.… Who am I to come in and say ‘Let's think about this in this way’?… The goal for them is to try and feel better about something and how we do that can look many different ways. (David).

While several participants considered general knowledge of challenges facing SMA was useful, they prioritised curiosity and avoided assuming a connection between adverse experiences and being a sexual minority, seeing which as ‘being broken and being something that wasn't right’: ‘When [she] talked to other people, they linked the two.… ‘That's why you're not interested in boys.’ She didn't feel like that was true at all and she felt like being gay was a positive thing’. (Ron) Moira described attending carefully to the young person's account rather than her own assumptions: ‘It's different if he's saying to me, ‘I'm getting homophobic abuse.’ That's quite different from me bringing those assumptions into the therapy room’. (Moira).

While many participants worked with SMA who were ‘confident in their identity’ and ‘don't necessarily want to explore as much [in therapy] because they've already normalised it’. (Ron), some noticed when young people arrived in crisis, with sexuality noted only as a background issue:

I've seen young people in the hospital after they've taken overdoses and sometimes a footnote on that referral will be ‘there may be questions about sexuality’. At that point… what I'm focused on is how are they feeling right now and how we get to this point. (Ron).

#### Facilitating conversations about sexuality

Regarding discovering a client's sexuality, many participants worked with SMA who ‘you don't have to ask. They'll tell you’ (Ron). Some became aware indirectly from referrals or parents. Meanwhile, some noticed SMA may first ‘test out [their] reactions’ (Twyla):

She would drop things… like talking about a [female] best friend and… describing them as sexy quite early on. I thought they were little tests.… I think she wanted to make it explicit, but she wasn't able to directly say ‘I'm gay or I like girls’. (Twyla).

Some participants mentioned that SMA may ‘need the therapist to broach [the topic of sexuality]’ (David), such as creating a space for conversations by asking questions that acknowledge sexual diversity: ‘I'd say, “Do you have a boyfriend or girlfriend?” depending on their age’ (Stevie). Others responded to cues or hesitation with attunement and reassurance:

I'd been able to name with them that I was picking up that there was something that they wanted to say and that when they wanted to talk about whatever it was, I would be there to listen and help them to make sense of it.… I've reiterated confidentiality if it was fear of [me having to let others know]. (Jocelyn).

Some participants mentioned experiencing positive emotions when SMA decided to share their concerns about sexuality, describing a ‘finally’ feeling. While most acknowledged that the ‘courage of being able to even say some of the words out loud’ (David), all said they would respond in a ‘no big deal’ (Ron) manner so SMA may experience their sexuality as ‘normal and nothing to be ashamed about’ (Stevie).

Considering their role is to ‘guide discoveries to help [SMA] figure out something… adults in [their] life don't always help with’ (David), most participants acknowledged that sexuality is ‘emerging’ in early adolescence and SMA may experience confusion and not be able to articulate their feelings: ‘[He said,] “I can't tell you what I'm confused about or how I'm confused about it but there's something”’ (David). David shared an account of supporting a 17‐year‐old teenager to explore the meaning of his feelings:

He was having thoughts of attraction with males, but also… with females, and some… anxious behaviours around men in school, but not quite sure why.… ‘Was it a place of actually I'm intimidated around other men? Or is it I'm attracted to other men, and this is actually something about my sexual attraction?’ (David).

Meanwhile, all participants reflected on how they used curiosity and acceptance to facilitate exploration and conversations:

[I was] really interested in how he was experiencing what he was experiencing… that somebody was interested in him, both from this person that he was having this relationship with, but also from me… that he was of interest and of value, and that this experience was of interest and of value. (Moira).

Anticipating potential impacts of stigma, some described the relational healing of being accepted in therapy: ‘If the therapist can offer something that's really healing as a response to [disclosure], the young person [may] take that outside of the therapy room and there's a “maybe not everyone will reject this”’. (David) Most participants also cautioned against rushing: ‘You have to stay alongside the process with them. You rush ahead, you lose them. There needs to be an openness and pace modelled to this’ (David).

#### Commitment to ongoing learning and humility

Some participants expressed shared humility and curiosity, especially regarding areas where their formal training was lacking: ‘Everything I learnt; I learnt through people’. (Jocelyn) They described learning directly from young people, and from being open to feedback: ‘If I don't get it, I want you to help me to’. (Stevie) Some questioned how well generalised knowledge prepared them for the variability of real experiences: ‘They're all going to be individual, aren't they? What's useful is… being able to bring some of that [knowledge] in… without being categorical about it, and [getting] out of it when it doesn't fit’ (Moira).

Participants who predominately worked indirectly with young people shared a desire to understand directly from SMA: ‘We're often grown‐ups talking about adolescents. I really want to know… what it is like for an adolescent in this day and age who identifies as a sexual minority from their experience’ (Twyla). David reflected on the benefits and risks of shared identity, noting that lived experience can deepen empathy but may create risks if the therapist is still processing their own identity.

#### Systemic collaboration beyond individual therapy

Many participants reflected on the potential impact psychological professionals could have beyond individual therapy. Several noted therapy alone may be insufficient if young people still face stigma in their everyday environment: ‘If they're being teased or bullied, [therapy] is going to be less effective’. (Moira) Ron described supporting a young person and their family together, while Jocelyn spoke about initiating more dialogue with colleagues in health care settings to support open conversations around sexuality.

While many participants argued that sexual identity labels are ‘rigid’ and tend to ‘fit people into boxes’ (Ron), some noticed others making assumptions about SMA's sexualities. Jocelyn shared a case where an adolescent's carers had labelled them ‘gay’ in their care plan after hearing they were attracted to someone of the same sex. In therapy, the young person said: ‘I don't know if I am gay… I'm not sure yet’. Jocelyn reflected, ‘People might get themselves in a little bit of a stuck position because they're trying to be an ally but actually might not really be listening to the young person’.

This reinforced participants' concern about premature labelling and the need for adults across systems to remain curious and responsive. Twyla echoed this by emphasising the value of working more closely with schools: ‘We can embed ourselves more in schools and with teachers so that we can really understand what happens and how we can have a positive impact on eradicating [negative experiences] where possible’.

### Wider socio‐political tensions influence how therapists navigate identity‐related work

This final theme introduces an overarching structural force that acts upon the entire therapeutic system. It explores how macro‐level professional and political dynamics exert a powerful causal influence on the micro‐level of the therapeutic space, constraining the therapist's agency and threatening to limit the narrative possibilities available to the young person.

#### Navigating professional polarisation and discourse tensions

Several participants described the current socio‐political climate within the psychological profession as increasingly polarised, generating anxiety around expressing views. Jocelyn highlighted a ‘polarisation of views within… and between professions’, leading to ‘fear of being ‘cancelled’ [or] criticised, [especially when] using the “wrong language”’. Moira similarly described a feeling of ‘you're not allowed to talk about that’, while Alexis said she often felt ‘disempowered’ when faced with what she perceived as monolithic expectations within the ‘LGBT community’. Social media was seen to exacerbate this anxiety:

Sometimes [people share] ‘my therapist said this really terrible thing to me’, and they go viral.… You're working in a way that feels sometimes quite defensive… not just because it could damage this relationship, but [because] it could… have wider consequences. (Alexis).

Several participants expressed concern that polarisation limited ‘open, free, honest discussions’ (Jocelyn). Moira noted that colleagues ‘use a lot of energy’ trying not to be judged, often shaping what they say to ‘fit’ rather than reflect their true views. Alexis likened this to putting on a ‘filter’ to engage professionally. Moira added: ‘There's too much of our profession seeing ourselves as activists first and therapist second… [A] fixed view of what's right and wrong [could be harmful if taken] into the therapy room and put on clients’. David emphasised the need for therapy to offer a space separate from prevailing socio‐political currents: ‘[Therapy] has to be exempt from the socio‐political world at times and… position itself almost side by side’.

Concerned about ‘echo chambers’, some participants called for professional spaces where complexity could be held without judgement, where individuals would ‘not just have permission, [but] be encouraged to talk about… current societal trends’ (Moira). Jocelyn described this as requiring ‘brave’ individuals willing to tolerate discomfort and hold nuance:

There need to be people who are brave enough to be able to put themselves forward and have nuanced conversations where they're aware of potential pulls to try to argue against and actually be able to hold the different perspectives but also think them through…. Often, we find that there's more commonality than we think within views. (Jocelyn).

#### Complexities and concerns around gender identity and SMA wellbeing

Some participants raised concerns about the conflation of sexuality and gender identity. Jocelyn reflected that, ‘There's a real fear of being able to talk openly about things around sexuality and in particular gender’. Stevie added, ‘Particularly around gender, people feel really deskilled’.

Alexis spoke about her clinical experiences supporting ‘de‐transitioners’—individuals who had reversed or discontinued medical or social transition. She noted that many eventually identified as lesbian or gay, raising concerns that premature transition might mask same‐sex attraction: ‘It's really difficult to predict the scale of things, but… it's already happening’.

Moira shared a worry that some SMA might internalise harmful societal messages that being gay is less acceptable than being transgender:

For young gay people [whose] peer group or family feel more comfortable with them being trans‐identified and being in a heterosexual relationship than being in a homosexual relationship.… I think we've got a real problem… They get pulled into, ‘Actually, it's better to alter my body than to be gay’. (Moira).

Participants stressed the importance of being able to engage in these complex and evolving conversations without fear of retribution, in order to support SMA thoughtfully, ethically and without eclipsing the young person's own narrative.

## DISCUSSION

The present study explored psychological therapists' experiences working with sexual minority adolescents (SMA) and factors facilitating or hindering their work within the current socio‐political landscape. This analysis identifies a central insight: participants emphasised the primacy of relational process in navigating the complexity of identity. Framed through a critical realist lens, this prioritisation can be understood as a mechanism to establish a *relational shield* against conflicting structural forces, protecting the adolescent's narrative from being eclipsed by external agendas.

These dynamics are captured in three interrelated themes. The first theme, *psychological therapists' experience of the socio‐environmental forces shaping adolescent sexuality*, establishes the layered contexts shaping young people's developing sexuality. In response, the second theme, *psychological therapists strive to offer attuned, responsive therapy*, reflects the clinical mechanism of the shield. Finally, the third theme, *wider socio‐political tensions influence how therapists navigate identity‐related work*, highlights how these external forces threaten the integrity of this protected space.

A key observation within the socio‐environmental context (Theme 1) was the participants' perception that younger adolescents seem to struggle to articulate their feelings of attraction. While resonating with classic developmental frameworks (Cass, 1979) and findings that identity consolidation typically lags behind attraction (Hall, Dawes, & Plocek, 2021), other research suggests this is not simply a ‘struggle to articulate’. Stewart et al.'s (2019) longitudinal study shows that adolescence is characterised by sexual fluidity and frequent discordance between identity, attractions and behaviours. These patterns suggest that adolescents' uncertainty may reflect negotiation of multiple, shifting identity possibilities. This process is also shaped by wider social change, with younger cohorts exposed to diverse identities moving through milestones more rapidly (Hall, Dawes, & Plocek, 2021). Therapists' observations may point to underlying mechanisms in which adolescents attempt to map felt experience onto culturally available identity categories that are themselves in flux.

This developmental uncertainty is compounded by emotional processes, particularly shame‐based distress. Participants observed that older adolescents' self‐consciousness and heightened sensitivity to peer evaluation can amplify internalised stigma (Reitz et al., 2014; Russell & Fish, 2019). Importantly, therapists emphasised that proximal influences such as family and peer reactions appeared to have a stronger impact on adolescents' self‐acceptance than broader societal attitudes (Bhugra et al., 2022; Chiang et al., 2019; Heiden‐Rootes et al., 2021). This was evident in their observations; for instance, one participant was surprised to find significant self‐hatred and shame even in a supposedly accepting community. This aligns with professional guidance emphasising these immediate interpersonal responses as key determinants of psychological wellbeing. The BPS (2024) notes the ‘lasting impact’ of proximal rejection, while Barker (2023) highlights that ‘access to supportive communities’ acts as a ‘vital buffer’ against distress (p.15).

In addition to these established social pressures, a novel insight regarding Theme 1 was participants' observation that heterosexuality is sometimes framed as ‘uncool’, creating peer pressure to identify as a sexual minority. While this may reflect the participants' assumptions about identity formation, it aligns with Amos et al.'s (2025) findings on the tensions in sexual minority in‐groups. They argue that the establishment of a ‘culture of queerness’ to resist heteronormativity can inadvertently create new exclusionary pressures. This is supported by their finding that ‘othering’ also occurred within this cultural space. This tension disadvantages those perceived as ‘too heteronormative’, such as a bisexual person with an opposite‐sex partner. Viewing heterosexuality as ‘uncool’ can therefore be understood as an unintended consequence of this in‐group formation, creating alternative social pressures that complicate identity navigation for all young people. While research typically focuses on rejection from heteronormative systems (e.g. Baams et al., 2020), this highlights the nuance of intra‐group tensions. Importantly, however, this must be balanced with observations that many SMA felt secure and confident in their identities (Pellicane et al., 2021; Wilson & Cariola, 2020).

Notwithstanding these peer dynamics, the wider structural context continues to enforce stigma against sexual minorities. The psychological impact of these combined pressures was evident in participants' discussions of internalised stigma. This, defined by Meyer (2003) as the ‘direction of negative social attitudes toward the self’ (p. 690), highlights a powerful real causal influence. Drawing on Diamond and Alley's (2022) idea of insufficient social safety, this stigma functions as a relational threat. It results in young people internalising the pervasive message that ‘heterosexuality is how you should be, and anything other is wrong’ (Amos et al., 2025, p. 16). Recognising that individual therapy alone may be insufficient given persistent stigma, participants strove to extend support systemically, for instance through family work or dialogues with health care colleagues. This reflects therapists' attempts to exercise agency within the relational and structural constraints they identified, extending the therapeutic frame to help create an environment where adolescents can re‐author their story.

Complementing these systemic efforts, participants emphasised the *agential striving* (Theme 2) required to keep adolescents in the ‘driver's seat’ regarding their identity. They described a deliberately client‐led approach, broaching sexuality only when relevant and avoiding the imposition of external conceptual frameworks. As one therapist noted: ‘I'm a political person but my politics shouldn't be infiltrating my therapy space. When our ideologies come into the room, we don't leave space for that person's story’. This sentiment reflects a recognition that societal narratives, whether affirmative or pathologising, can inadvertently narrow the space for a young person's own meaning‐making. This active restraint aligns with the concept of *negative capability* (Cornish, 2011), the skill to remain in uncertainty without reaching for premature resolution. Therapists therefore sought to protect that space, expressing a specific concern that premature categorisation, whether by carers, systems or the therapist themselves, could eclipse the adolescent's emerging agency.

This client‐led stance was particularly evident in how participants navigated the use of identity labels. Participants observed some adolescents ‘like having a label to describe how they see themselves’, which could foster a sense of belonging. This aligns with research confirming that conventional sexual identity labels can hold inherent value and utility for many young people in classifying their experiences (Glover et al., 2009). Conversely, while acknowledging that labelling is a common human process, participants also saw clients reject fixed categories. For instance, one young person insisted they were ‘just undefinable’ when offered the label ‘pansexual’. This mirrors findings that a subset of young people prefers experiential descriptions over fixed identities (Glover et al., 2009), reinforcing the recognition that sexual minorities are not a homogenous group (Bhugra et al., 2022). Therapists responded by attending closely to the meaning a label held for each young person, treating labels as flexible resources rather than developmental endpoints.

This focus on the adolescent's narrative was supported by a reliance on core clinical skills. Given limited formal training in psychosexual development (Ho et al., 2023), participants navigated the complexity of identity work by using existing conceptual frameworks cautiously and prioritising curiosity, compassion and relational safety, centred on attunement (Bruce et al., 2015; Kelley, 2015; King, 2008). However, this self‐appraisal stands in tension with findings that young people often view a lack of specialist knowledge as a barrier to support, reporting clinicians were not ‘adequately trained’ (Higgins et al., 2021, p. 61).

This gap may reflect the inherent challenge of defining ‘competence’ for working with a heterogeneous population, particularly during adolescence. As Dullius et al. (2019) noted in their systematic review of health care professionals' competencies, there is ‘no indication in the literature about which are the competencies to be developed’ (p. 8). This ambiguity aligns with existing guidance for working with sexual minority adults (Barker, 2023; BPS, 2024), which warns against generalised assumptions, emphasising instead that individuals have vastly different needs and intersections of identity. For adolescents, this heterogeneity is further compounded by developmental fluidity and intense social pressures. Consequently, participants resolved this tension by privileging the adolescent's specific lived experience over static content. By relying on relational safety, a primary vehicle for therapeutic change (Wampold & Imel, 2015), they navigated an evolving identity that no single manualised framework could fully capture.

From this perspective, these findings suggest that relational safety functions as more than a clinical tool; it appears to act as a relational shield against conflicting structural forces. As identified in Theme 1, adolescents navigate intense peer and social pressures regarding identity. Simultaneously, Theme 3 reveals that therapists themselves must navigate professional polarisation and rigid societal narratives. Consequently, the agential striving described in Theme 2 acts as the clinical mechanism to withstand these pressures, achieved by using supervision and reflexivity to mitigate personal biases, ensuring the young person's emerging narrative is never eclipsed by external agendas.

This active monitoring of personal pulls relies on what Archer (2003) terms the *internal conversation*, the continuous reflexive process through which agents deliberate on their personal concerns in relation to their social circumstances (as cited in Alderson, 2021). However, maintaining this protective stance is complicated by the reality that, as Alderson (2021) asserts, the positivist ideal of value‐free neutrality is impossible when values pervade social and clinical facts. This tension frequently manifests as difficulty for therapists in holding a nuanced dialogue. For example, while professional bodies like the BPS (2024) rightly encourage psychologists to ‘work on an institutional level’ to ‘reduce social stigma’ (p. 8), this study reveals a significant barrier to this aspiration. Participants described intense professional polarisation, which, in their view, creates a climate of fear, stifling open discussion and hindering engagement in this essential systemic role.

In the participants' accounts, a key contributor to this polarisation was the complex discourse surrounding the conflation of sexuality and gender identity. Participants voiced concerns similar to those in the Independent Review of Gender Identity Services (Cass, 2024), which noted cultural contexts where ‘being transgender is seen as preferable to being same‐sex attracted’ and reported that some young people identified as transgender before recognising same‐sex attraction (p. 119). The Cass Review has become a focal point for intense professional disagreement, where the divergence of scholarly opinion contributes directly to the polarisation described by participants. The debate includes both methodological critiques against the Review (e.g. McNamara et al., 2024; Noone et al., 2025) and defences of its findings and cautionary approach against those critiques (e.g. McDeavitt et al., 2025). Crucially, this analysis does not aim to adjudicate these debates, but to illustrate how professional polarisation may exert a structural pressure constraining the therapist's capacity to think and speak freely.

This constraint is clinically significant because, as established in the literature, sexual minority individuals often explore different gender expressions, reflecting higher levels of gender non‐conformity (Thoma et al., 2021). Without a protected space to explore this overlap, there is a risk that normative gender non‐conformity may be conflated with gender‐related distress. This poses a clinical challenge, as therapists may struggle to distinguish these developmental pathways when structural pressures make open discussion feel risky. In such a volatile professional climate, the necessary clinical capacity to hold uncertainty becomes significantly harder to sustain. This anxiety illustrates how macro‐level political structures may exert a real causal power on the therapy relationship. By creating a context where shifting political values complicate consistent practice, this structural pressure manifests as a challenging *malign tendency* within the social field (see Alderson, 2021).

Reflecting this mechanism, participants' concerns highlight how polarisation risks leaving minority sexuality overlooked. In response, therapists balance attuned, client‐led support with the ethical responsibility to critically reflect on their role within societal narratives. They strive to maintain therapeutic openness and safeguard adolescents whose minority sexuality may be overshadowed by gender‐focused discourse, in line with guidance to ‘explore, compassionately challenge, and hold uncertainty’ (BPS, 2024, p. 6).

These themes, viewed through a critical realist lens, illustrate the interplay of structural forces, relational processes and agential mediation. They highlight how developmental complexity, shifting peer cultures and socio‐political tensions shape therapists' ability to support sexual minority adolescents, influencing what facilitates or hinders their work and how they navigate the therapeutic process.

### Clinical implications

This study offers several implications for professional practice, particularly given the identified gap in UK clinical guidance for working with this population. While these findings reflect the self‐appraisals of the seven therapists who participated in this study, which must be balanced against client reports of unmet needs (e.g. Higgins et al., 2021), their focus on relational process aligns closely with existing adult principles, suggesting these skills are foundational.

Reflecting the central insight of this study, effective practice appears to function as a relational shield. Given that navigating identity development takes time (Hall, Dawes, & Plocek, 2021), exploratory work requires pacing that maintains the integrity of the therapeutic process. Therapists can support this by offering a non‐judgemental and confidential space, using negative capability (Cornish, 2011) to tolerate the anxiety of uncertainty without reaching for premature resolution. This approach relies on attunement and alliance‐building (Wampold & Imel, 2015), rather than a mastery of generalised knowledge.

To maintain this shield, training and supervision should therefore prioritise the development of the internal conversation, helping clinicians distinguish between the adolescent's needs and their own political or moral pulls. By rigorously monitoring when and how sexuality is relevant, therapists can ensure the young person's narrative is never eclipsed by external agendas, while remaining attentive to the nuances and fluidity of sexuality (Woodford et al., 2018).

Furthermore, formulation should emphasise a structural understanding of distress. Minority stress (Meyer, 2003) should be understood as a model of socio‐political disadvantage requiring systemic advocacy alongside individual resilience (Frost & Meyer, 2023). Psychological professionals can promote social safety within health care and education, for instance, by advocating against premature framing or rigid categorisation, helping systems tolerate complexity to ensure a young person's experience is not automatically ascribed to a fixed identity category or overshadowed by existing narratives.

Finally, these principles depend on the professional climate. The identified malign tendency of polarisation may exert pressure that can constrain therapeutic agency. Reducing this structural weight is therefore clinically crucial. Cultivating respectful dialogue is essential to safeguard the therapeutic work. As psychological therapists who support others to explore complexity (Bromberg, 2022), the profession must model the capacity to tolerate ambiguity. While ‘people with differing views can find it uncomfortable to sit together in the same room’ (Cass, 2024, p. 60), discomfort is not a reason to avoid constructive discussions. Training communities, services and professional networks share a responsibility to foster environments where open, nuanced conversations are encouraged, ensuring that the fear of ‘getting it wrong’ does not silence the necessary exploration.

### Limitations

This study contains several practical and theoretical shortcomings. First, the findings are based on a small sample. As Alderson (2021) notes, in‐depth analysis of complex mechanisms can be achieved through intensive analysis of a few cases rather than large samples. While this dataset afforded the explanatory power valued by critical realism, the findings reflect the specific experiences of these seven individuals and cannot be generalised to the profession at large, warranting further exploration and theoretical testing.

Second, although eligibility criteria ensured participants had experience supporting SMA, this may have excluded cases where adolescents did not discuss their sexuality, either because it was not relevant or because they did not feel able to disclose it. Furthermore, variation in participant characteristics may have influenced the data. For example, clinical experience varied widely, and only two participants identified as sexual minorities. This implies that the majority of the data reflects a heterosexual perspective on minority experience. This ‘outsider’ lens may have influenced interpretations of specific dynamics, such as the complexity of peer pressure regarding sexual fluidity.

Third, the findings are context‐specific to the particularly polarised UK socio‐political climate, which Cass (2024, p. 20) describes as ‘toxic’. Crucially, this polarisation appears to centre not on sexuality itself, but on the closely associated and frequently conflated topic of gender identity. This specific context not only limits the generalisability of the insights to other settings but also likely influenced sample selection. The polarised nature of this associated topic may have disproportionately attracted therapists with strong or settled views on either side of the debate, while potentially excluding those who feel ambivalent, anxious or silenced by these tensions.

Finally, only two participants were employed within NHS services, which limits representativeness from statutory settings. Consequently, findings may not fully capture the NHS clinical realities, such as high demand and clinical risk (Dutton et al., 2023).

### Future research

This study highlights several areas for further investigation. While literature has explored sexual minority clients' experiences of therapy (e.g. Higgins et al., 2021; Israel et al., 2008), future research could specifically examine the adolescent perspective on the ‘shielding’ function of the therapist, exploring what young people find helpful regarding the management of identity exploration, particularly in relation to labelling and pacing. Additionally, considering the findings that some young people struggle to accept their sexual feelings and attractions, future studies could examine factors from young people's perspectives that reduce self‐rejection and promote self‐acceptance within the evolving social landscape.

Finally, participants expressed concern that contemporary social narratives may lead adolescents to internalise the belief that ‘it's better to alter [their] body than to be gay’, potentially overshadowing same‐sex attraction. While acknowledging that these concerns reflect the perspectives of a small, predominantly heterosexual sample, this possibility aligns with observations that some adolescents who undergo interventions for gender‐related distress do later identify as sexual minorities (Cass, 2024). This highlights a need to investigate the role of wider cultural influences on identity development, particularly considering the potential conflation of sexuality and gender identity. However, these observations should be contextualised within the broader evidence base, which remains characterised by uncertainty. Current estimates vary widely depending on definitions, ranging from <1% for surgical regret to approximately 7–10% for the discontinuation of transition‐related care (Hall, Mitchell, & Sachdeva, 2021; Irwig, 2022), though high rates of disengagement suggest these figures may be incomplete (Hall, Mitchell, & Sachdeva, 2021). Consequently, future research should explore how clinicians navigate this uncertainty, specifically how their understandings of these complex identity trajectories and the socio‐political forces shaping them influence formulation, decision‐making and therapeutic support.

## CONCLUSION

This study, framed through a critical realist lens, explored the experiences of seven UK‐based psychological therapists supporting SMA. The analysis identified three underlying causal social mechanisms shaping their experiences: the influence of socio‐environmental factors on sexuality development, therapists' efforts to provide attuned and helpful therapy, and the enactment of socio‐political tensions within the profession.

The participants' accounts illustrated how socio‐political forces shape therapeutic engagement. The findings suggest the importance of meeting adolescents with curiosity and care, acting as a shield against the imposition of societal frameworks. While highlighting the participants' commitment for systemic change, the study also brought to light concerns and tensions they experience, which are often left unspoken.

The analysis points to the need for profession‐wide engagement in open, nuanced dialogue to navigate this complexity. Ultimately, the findings suggest that therapy functions as a mediating space, shielding developing adolescents from polarisation and stigma. This reinforces a shared professional tenet that the protective integrity of the therapeutic process is often more healing than a premature focus on content‐driven outcomes.

## AUTHOR CONTRIBUTIONS

**Paris Young:** Conceptualization; methodology; investigation; formal analysis; project administration; writing – original draft; writing – review and editing. **Nargis Islam:** Supervision; writing – review and editing; methodology; conceptualization. **James Lea:** Supervision; methodology; writing – review and editing. **Anna Daiches:** Supervision; writing – review and editing; methodology; conceptualization.

## FUNDING INFORMATION

This research received no specific research grant from any funding agency in the public, commercial or not‐for‐profit sectors. The work was completed as part of the lead author's doctoral training programme in clinical psychology at Lancaster University, UK.

## CONFLICT OF INTEREST STATEMENT

The authors declare that they have no conflicts of interest.

## ETHICS STATEMENT

Ethical approval was obtained from the Faculty of Health and Medicine Research Ethics Committee at Lancaster University (FHM‐2022‐0932‐RECR‐2). Informed consent was obtained from all participants before the interviews.

## Supporting information


Data S1

Data S2

## Data Availability

The data that support the findings of this study are available from the corresponding author upon reasonable request.

## References

[papt70051-bib-0001] Alderson, P. (2021). Critical realism for health and illness research. Bristol University Press.

[papt70051-bib-0002] Amos, R. , Manalastas, E. J. , White, R. , Bos, H. , & Patalay, P. (2020). Mental health, social adversity, and health‐related outcomes in sexual minority adolescents: A contemporary national cohort study. The Lancet Child & Adolescent Health, 4(1), 36–45. 10.1016/S2352-4642(19)30339-6 31753807

[papt70051-bib-0003] Amos, R. , White, R. , Patalay, P. , & Donnellan, W. (2025). The dynamic identity formation of sexual minority adolescents: A constructivist grounded theory of adversity and resilience. Journal of Homosexuality, 73, 1–27. 10.1080/00918369.2025.2493150 40445090

[papt70051-bib-0004] APA . (2021). APA resolution on sexual orientation change efforts . https://www.apa.org/about/policy/resolution‐sexual‐orientation‐change‐efforts.pdf

[papt70051-bib-0005] Archer, M. S. (2003). Structure, agency and the internal conversation. Cambridge University Press.

[papt70051-bib-0006] Ayhan, C. H. B. , Bilgin, H. , Uluman, O. T. , Sukut, O. , Yilmaz, S. , & Buzlu, S. (2020). A systematic review of the discrimination against sexual and gender minority in health care settings. International Journal of Health Services, 50(1), 44–61. 10.1177/0020731419885093 31684808

[papt70051-bib-0007] Baams, L. , Kiekens, W. J. , & Fish, J. N. (2020). The rejection sensitivity model: Sexual minority adolescents in context. Archives of Sexual Behavior, 49(7), 2259–2263. 10.1007/s10508-019-01572-2 31664554 PMC7497447

[papt70051-bib-0008] BACP . (2022). Memorandum of understanding on conversion therapy in the UK . https://www.bacp.co.uk/events‐and‐resources/ethics‐and‐standards/mou/

[papt70051-bib-0009] Bafiti, T. , Viou, M. , & Tarasis, P. (2019). “Stepping up the ladder in safety”: An interpretative phenomenological analysis of how LGB clients experience their therapists' sexual orientation. The European Journal of Counselling Psychology, 7(1), 211–223. 10.5964/ejcop.v7i1.139

[papt70051-bib-0010] Bandera, G. (2023). Which countries impose the death penalty on gay people? Fair Planet . https://www.fairplanet.org/story/death‐penalty‐homosexualty‐illegal/

[papt70051-bib-0011] Barberá, P. (2020). Social media, echo chambers, and political polarization. In N. Persily & J. A. Tucker (Eds.), Social media and democracy: The state of the field, prospects for reform (pp. 34–55). Cambridge University Press.

[papt70051-bib-0012] Barker, M.‐J. (2023). Gender, sexual, and relationship diversity (GSRD) . Good Practice across the Counselling Professions 001. British Association for Counselling and Psychotherapy.

[papt70051-bib-0013] Bhaskar, R. (2008). A realist theory of science. Routledge.

[papt70051-bib-0014] Bhaskar, R. , & Hartwig, M. (2016). Enlightened common sense: The philosophy of critical realism. Routledge.

[papt70051-bib-0015] Bhugra, D. , Killaspy, H. , Kar, A. , Levin, S. , Chumakov, E. , Rogoza, D. , Harvey, C. , Bagga, H. , Owino–Wamari, Y. , Everall, I. , Bishop, A. , Javate, K. R. , Westmore, I. , Ahuja, A. , Torales, J. , Rubin, H. , Castaldelli‐Maia, J. , Ng, R. , Nakajima, G. A. , … Ventriglio, A. (2022). IRP commission: Sexual minorities and mental health: Global perspectives. International Review of Psychiatry, 34, 171–199. 10.1080/09540261.2022.2045912 36151836

[papt70051-bib-0016] Bochicchio, L. , Reeder, K. , Ivanoff, A. , Pope, H. , & Stefancic, A. (2020). Psychotherapeutic interventions for LGBTQ + youth: A systematic review. Journal of LGBT Youth, 19, 152–179. 10.1080/19361653.2020.1766393

[papt70051-bib-0017] Bond, B. J. (2014). Sex and sexuality in entertainment media popular with lesbian, gay, and bisexual adolescents. Mass Communication and Society, 17(1), 98–120. 10.1080/15205436.2013.816739

[papt70051-bib-0018] Braun, V. , & Clarke, V. (2006). Using thematic analysis in psychology. Qualitative Research in Psychology, 3(2), 77–101. 10.1191/1478088706qp063oa

[papt70051-bib-0019] Braun, V. , & Clarke, V. (2022). Thematic analysis: A practical guide. SAGE Publications.

[papt70051-bib-0020] British Psychological Society . (2024). Guidelines for psychologists working with gender, sexuality and relationship diversity (2nd ed.). British Psychological Society. 10.53841/bpsrep.2024.rep129b

[papt70051-bib-0021] Bromberg, P. M. (2022). Psychotherapy as the art of uncertainty. Contemporary Psychoanalysis, 58, 176–201. 10.1080/00107530.2022.2145975

[papt70051-bib-0022] Brooks, V. R. (1981). Minority stress and lesbian women. Lexington Books.

[papt70051-bib-0023] Bruce, D. , Harper, G. W. , & Bauermeister, J. A. (2015). Minority stress, positive identity development, and depressive symptoms: Implications for resilience among sexual minority male youth. Psychology of Sexual Orientation and Gender Diversity, 2(3), 287–296. 10.1037/sgd0000128 26478901 PMC4603569

[papt70051-bib-0024] Carrington, M. , & Sims, M. (2024). How can counselling training courses better prepare their trainee therapists to work with LGBTQ+ clients? Counselling and Psychotherapy Research, 24(2), 513–523. 10.1002/capr.12684

[papt70051-bib-0025] Cass, H. (2024). Independent review of gender identity services for children and young people: Final report . https://cass.independent‐review.uk/wp‐content/uploads/2024/04/CassReview_Final.pdf 10.1192/bjp.2024.162 39237983

[papt70051-bib-0026] Cass, V. C. (1979). Homosexual identity formation: A theoretical model. Journal of Homosexuality, 4(3), 219–235. 264126 10.1300/J082v04n03_01

[papt70051-bib-0027] Chiang, S.‐Y. , Fenaughty, J. , Lucassen, M. F. G. , & Fleming, T. (2019). Navigating double marginalisation: Migrant Chinese sexual and gender minority young people's views on mental health challenges and supports. Culture, Health & Sexuality, 21(7), 807–821. 10.1080/13691058.2018.1519118 30409106

[papt70051-bib-0028] Cornish, S. (2011). Negative capability and social work: Insights from Keats, bion and business. Journal of Social Work Practice, 25(2), 135–148.

[papt70051-bib-0029] Diamond, L. M. , & Alley, J. (2022). Rethinking minority stress: A social safety perspective on the health effects of stigma in sexually‐diverse and gender‐diverse populations. Neuroscience and Biobehavioral Reviews, 138, 104720. 10.1016/j.neubiorev.2022.104720 35662651

[papt70051-bib-0030] Dullius, W. R. , Martins, L. B. , & Cesnik, V. M. (2019). Systematic review on health care professionals' competencies in the care of LGBT+ individuals. Estudos de Psicologia (Campinas), 36, 1–14. 10.1590/1982-0275201936e180171

[papt70051-bib-0031] Dutton, B. , Humphrey, N. , & Qualter, P. (2023). Getting the pieces to fit: NHS and third sector collaboration to enhance crisis mental health service provision for young people. BMC Health Services Research, 23(1), 307. 10.1186/s12913-023-09198-w 36997929 PMC10061406

[papt70051-bib-0032] Fletcher, A. J. (2017). Applying critical realism in qualitative research: Methodology meets method. International Journal of Social Research Methodology, 20(2), 181–194. 10.1080/13645579.2016.1144401

[papt70051-bib-0033] Frost, D. M. , & Meyer, I. H. (2023). Minority stress theory: Application, critique, and continued relevance. Current Opinion in Psychology, 51, 101579. 10.1016/j.copsyc.2023.101579 37270877 PMC10712335

[papt70051-bib-0034] Fryer, T. (2022). A critical realist approach to thematic analysis: Producing causal explanations. Journal of Critical Realism, 21(4), 365–384. 10.1080/14767430.2022.2076776

[papt70051-bib-0035] Gilbey, D. , Mahfouda, S. , Ohan, J. , Lin, A. , & Perry, Y. (2020). Trajectories of mental health difficulties in young people who are attracted to the same gender: A systematic review. Adolescent Research Review, 5(3), 281–293. 10.1007/s40894-019-00128-8

[papt70051-bib-0036] Glover, J. A. , Galliher, R. V. , & Lamere, T. G. (2009). Identity development and exploration among sexual minority adolescents: Examination of a multidimensional model. Journal of Homosexuality, 56(1), 77–101. 10.1080/00918360802551555 19197644

[papt70051-bib-0037] Graham, T. C. (2019). Conversion therapy: A brief reflection on the history of the practice and contemporary regulatory effects. Creighton Law Review, 52, 419.

[papt70051-bib-0038] Hall, R. , Mitchell, L. , & Sachdeva, J. (2021). Access to care and frequency of detransition among a cohort discharged by a UK national adult gender identity clinic: Retrospective case‐note review. BJPsych Open, 7(6), e184. 10.1192/bjo.2021.1022 34593070 PMC8503911

[papt70051-bib-0039] Hall, W. J. , Dawes, H. C. , & Plocek, N. (2021). Sexual orientation identity development milestones among lesbian, gay, bisexual, and queer people: A systematic review and meta‐analysis. Frontiers in Psychology, 12, 753954. 10.3389/fpsyg.2021.753954 34777153 PMC8581765

[papt70051-bib-0040] Heiden‐Rootes, K. , Ross, K. , Moore, R. , Hasan, S. , & Gulotta, S. (2021). Freedom and struggling openly in psychotherapy: A qualitative inquiry with LGBQ young adults from religious families. Journal of Marital and Family Therapy, 47(1), 150–165. 10.1111/jmft.12442 32652676

[papt70051-bib-0041] Higgins, A. , Downes, C. , Murphy, R. , Sharek, D. , Begley, T. , McCann, E. , & Doyle, L. (2021). LGBT+ young people's perceptions of barriers to accessing mental health services in Ireland. Journal of Nursing Management, 29(1), 58–67. 33068465 10.1111/jonm.13186

[papt70051-bib-0042] Ho, J. K. Y. , O'Rouke, C. , Laville, A. , Chellingsworth, M. , & Callaghan, P. (2023). Clinician experiences on training and awareness of sexual orientation in NHS talking therapies Services for Anxiety and Depression. The Cognitive Behaviour Therapist, 16, e24. 10.1017/S1754470X23000181

[papt70051-bib-0043] Irish, M. , Solmi, F. , Mars, B. , King, M. , Lewis, G. , Pearson, R. M. , Pitman, A. , Rowe, S. , Srinivasan, R. , & Lewis, G. (2019). Depression and self‐harm from adolescence to young adulthood in sexual minorities compared with heterosexuals in the UK: A population‐based cohort study. The Lancet Child & Adolescent Health, 3(2), 91–98. 10.1016/S2352-4642(18)30343-2 30552054

[papt70051-bib-0044] Irwig, M. S. (2022). Detransition among transgender and gender‐diverse people—An increasing and increasingly complex phenomenon. The Journal of Clinical Endocrinology & Metabolism, 107(10), e4261–e4262. 10.1210/clinem/dgac356 35678284 PMC9516050

[papt70051-bib-0045] Israel, T. , Gorcheva, R. , Burnes, T. R. , & Walther, W. A. (2008). Helpful and unhelpful therapy experiences of LGBT clients. Psychotherapy Research, 18(3), 294–305. 10.1080/10503300701506920 18815981

[papt70051-bib-0046] Jaspal, R. , Lopes, B. , & Breakwell, G. M. (2023). Minority stressors, protective factors and mental health outcomes in lesbian, gay and bisexual people in the UK. Current Psychology, 42(28), 24918–24934. 10.1007/s12144-022-03631-9

[papt70051-bib-0047] Kahle, L. (2020). Are sexual minorities more at risk? Bullying victimization among lesbian, gay, bisexual, and questioning youth. Journal of Interpersonal Violence, 35(21–22), 4960–4978. 10.1177/0886260517718830 29294825

[papt70051-bib-0048] Kelleher, C. (2009). Minority stress and health: Implications for lesbian, gay, bisexual, transgender, and questioning (LGBTQ) young people. Counselling Psychology Quarterly, 22(4), 373–379. 10.1080/09515070903334995

[papt70051-bib-0049] Kelley, F. A. (2015). The therapy relationship with lesbian and gay clients. Psychotherapy, 52(1), 113–118. 10.1037/a0037958 25365154

[papt70051-bib-0050] Khanolkar, A. R. , Frost, D. M. , Tabor, E. , Redclift, V. , Amos, R. , & Patalay, P. (2023). Ethnic and sexual identity‐related inequalities in adolescent health and well‐being in a national population‐based study. LGBT Health, 10(1), 26–40. 10.1089/lgbt.2021.0473 36049061

[papt70051-bib-0051] Kinderman, P. (2014). The role of the psychologist in social change. International Journal of Social Psychiatry, 60(4), 403–405. 10.1177/0020764013491741 23788437

[papt70051-bib-0052] King, S. (2008). Exploring the role of counselor support: Gay, lesbian, bisexual, and questioning adolescents struggling with acceptance and disclosure. Journal of GLBT Family Studies, 4(3), 361–384. 10.1080/15504280802177599

[papt70051-bib-0053] Lin, C. , Moore, D. D. , Nylund, D. , & Espinoza, S. A. (2020). Clinical issues among Chinese gay men in counseling. Journal of LGBT Issues in Counseling, 14(1), 18–37. 10.1080/15538605.2020.1711290

[papt70051-bib-0054] Mathieson, F. , Garrett, S. , Stubbe, M. , Hilder, J. , Tester, R. , Fedchuk, D. , Dunlop, A. , & Dowell, A. (2023). Therapist voices on a youth mental health pilot: Responsiveness to diversity and therapy modality. International Journal of Environmental Research and Public Health, 20(3), 1834. 10.3390/ijerph20031834 36767203 PMC9914598

[papt70051-bib-0055] McDeavitt, K. , Cohn, J. , & Levine, S. B. (2025). Critiques of the cass review: Fact‐checking the peer‐reviewed and grey literature. Journal of Sex & Marital Therapy, 51(2), 175–199. 10.1080/0092623X.2025.2455133 39903043

[papt70051-bib-0056] McNamara, M. , Baker, K. , Connelly, K. , Janssen, A. , Olson‐Kennedy, J. , Pang, K. C. , Scheim, A. , Turban, J. , & Alstott, A. (2024). An evidence‐based critique of the Cass review on gender‐affirming care for adolescent gender dysphoria. Yale School of Medicine; Yale Law School. https://law.yale.edu/sites/default/files/documents/integrity_project_cass_review.pdf

[papt70051-bib-0057] Meyer, I. H. (2003). Prejudice, social stress, and mental health in lesbian, gay, and bisexual populations: Conceptual issues and research evidence. Psychological Bulletin, 129(5), 674–697. 10.1037/0033-2909.129.5.674 12956539 PMC2072932

[papt70051-bib-0058] Meyer, I. H. (2014). Minority stress and positive psychology: Convergences and divergences to understanding LGBT health. Psychology of Sexual Orientation and Gender Diversity, 1(4), 348–349. 10.1037/sgd0000070

[papt70051-bib-0060] Noone, C. , Southgate, A. , Ashman, A. , Quinn, É. , Comer, D. , Shrewsbury, D. , Ashley, F. , Hartland, J. , Paschedag, J. , Gilmore, J. , Kennedy, N. , Woolley, T. E. , Heath, R. , Biskupovic Goulding, R. , Simpson, V. , Kiely, E. , Coll, S. , White, M. , Grijseels, D. M. , … McLamore, Q. (2025). Critically appraising the cass report: Methodological flaws and unsupported claims. BMC Medical Research Methodology, 25(1), 1–18. 10.1186/s12874-025-02581-7 40348955 PMC12065279

[papt70051-bib-0061] ONS . (2023). Sexual orientation, UK: 2021 and 2022 . Census 2021. https://www.ons.gov.uk/peoplepopulationandcommunity/culturalidentity/sexuality/bulletins/sexualidentityuk/2021and2022#sexual‐orientation‐in‐the‐uk

[papt70051-bib-0062] Pachankis, J. E. , Mahon, C. P. , Jackson, S. D. , Fetzner, B. K. , & Bränström, R. (2020). Sexual orientation concealment and mental health: A conceptual and meta‐analytic review. Psychological Bulletin, 146(10), 831–871. 10.1037/bul0000271 32700941 PMC8011357

[papt70051-bib-0063] Pellicane, M. J. , Cooks, J. A. , & Ciesla, J. A. (2021). Longitudinal effects of social media experiences on depression and anxiety in LGB+ and heterosexual young adults. Journal of Gay & Lesbian Mental Health, 25(1), 68–93. 10.1080/19359705.2020.1776805

[papt70051-bib-0064] Reitz, A. K. , Zimmermann, J. , Hutteman, R. , Specht, J. , & Neyer, F. J. (2014). How peers make a difference: The role of peer groups and peer relationships in personality development. European Journal of Personality, 28(3), 279–288. 10.1002/per.1965

[papt70051-bib-0065] Rich, A. J. , Salway, T. , Scheim, A. , & Poteat, T. (2020). Sexual minority stress theory: Remembering and honoring the work of Virginia Brooks. LGBT Health, 7(3), 124–127. 10.1089/lgbt.2019.0223 32053038

[papt70051-bib-0066] Robinson, B. A. (2016). Heteronormativity and homonormativity. In N. Naples (Ed.), The Wiley Blackwell encyclopedia of gender and sexuality studies (pp. 1–3). Wiley. 10.1002/9781118663219.wbegss013

[papt70051-bib-0067] Rog, D. J. , Reidy, M. C. , Manian, N. , Daley, T. C. , & Lieberman, L. (2021). Opportunities for psychologists to enact community change through adverse childhood experiences, trauma, and resilience networks. American Psychologist, 76(2), 379–390. 10.1037/amp0000778 33734802

[papt70051-bib-0069] Russell, S. T. , & Fish, J. N. (2019). Sexual minority youth, social change, and health: A developmental collision. Research in Human Development, 16(1), 5–20. 10.1080/15427609.2018.1537772 31602178 PMC6786797

[papt70051-bib-0071] Scroggs, B. , & Vennum, A. (2021). Gender and sexual minority group identification as a process of identity development during emerging adulthood. Journal of LGBT Youth, 18(3), 287–304. 10.1080/19361653.2020.1722780

[papt70051-bib-0072] Shpigel, M. S. , & Diamond, G. M. (2014). Good versus poor therapeutic alliances with non‐accepting parents of same‐sex oriented adolescents and young adults: A qualitative study. Psychotherapy Research, 24(3), 376–391. 10.1080/10503307.2013.856043 24286222

[papt70051-bib-0073] Spittlehouse, J. K. , Boden, J. M. , & Horwood, L. J. (2020). Sexual orientation and mental health over the life course in a birth cohort. Psychological Medicine, 50(8), 1348–1355. 10.1017/S0033291719001284 31190681

[papt70051-bib-0074] Stewart, J. L. , Spivey, L. A. , Widman, L. , Choukas‐Bradley, S. , & Prinstein, M. J. (2019). Developmental patterns of sexual identity, romantic attraction, and sexual behavior among adolescents over three years. Journal of Adolescence, 77, 90–97. 10.1016/j.adolescence.2019.10.006 31693971 PMC6885553

[papt70051-bib-0075] Thoma, B. C. , Eckstrand, K. L. , Montano, G. T. , Rezeppa, T. L. , & Marshal, M. P. (2021). Gender nonconformity and minority stress among lesbian, gay, and bisexual individuals: A meta‐analytic review. Perspectives on Psychological Science, 16(6), 1165–1183. 10.1177/1745691620968766 33645322 PMC7646043

[papt70051-bib-0076] Wampold, B. E. , & Imel, Z. E. (2015). The great psychotherapy debate: The evidence for what makes psychotherapy work (2nd ed.). Routledge/Taylor & Francis Group.

[papt70051-bib-0077] Wilson, C. , & Cariola, L. A. (2020). LGBTQI+ youth and mental health: A systematic review of qualitative research. Adolescent Research Review, 5(2), 187–211. 10.1007/s40894-019-00118-w

[papt70051-bib-0078] Wiltshire, G. , & Ronkainen, N. (2021). A realist approach to thematic analysis: Making sense of qualitative data through experiential, inferential and dispositional themes. Journal of Critical Realism, 20, 159–180. 10.1080/14767430.2021.1894909

[papt70051-bib-0079] Woodford, M. R. , Joslin, J. Y. , & Marshall, Z. (2018). Starting with LGB(T): Methodological considerations in quantitative gender and sexual identity research in higher education. In M. R. Woodford , J. Y. Joslin , & Z. Marshall (Eds.), Starting with gender in international higher education research: Conceptual debates and methodological considerations (pp. 98–125). Routledge. 10.4324/9781315100906-7

[papt70051-bib-0080] Żerebecki, B. G. , Opree, S. J. , Hofhuis, J. , & Janssen, S. (2021). Can TV shows promote acceptance of sexual and ethnic minorities? A literature review of television effects on diversity attitudes. Sociology Compass, 15(8), 1–16. 10.1111/soc4.12906

