# Supplementary material for: Supporting sexual minority adolescents: A critical realist thematic analysis of psychological therapists' experiences
Source: Psychol Psychother. 2026 Mar 5;99(2):684–704. doi: 10.1111/papt.70051 (PMC13162195; doi:10.1111/papt.70051)
Supplement: Supplementary file 1 — Data S1 [file PAPT-99-684-s002.docx]

**Interview Question Guide**

This interview guide offers a typical structure and some questions and prompts (in italics) that may be asked during an interview. It offers a guide for the interviewer to explore topic areas that may help elicit information from the participant to answer the research question. This guide will not be strictly followed as additional questions will be adapted in response to the participant’s answers during the interview.

***Pre-interview Statements***

- In this interview, we will focus on your experience working with sexual minority adolescents (13 – 19) as a psychological therapist. However, their sexuality does not have to be a focus of your work. When you answer the following questions, I’d like you to think about a young person or a few young people you worked with who was of a sexual minority.
- We understand sharing personal experiences and thoughts can be difficult when it comes to discussing about sexuality and inclusivity. Sometimes, it can feel like there is a right way to say things or there are appropriate answers that fit a ‘party line’. Please be open and honest with your thoughts and feelings about the questions and answer as truthfully as you can. Your responses are protected by confidentiality and will not be in any way linked back to you.

***Interview Questions***

1. Without giving any identifiable information, could you tell me how you learnt about their sexuality? [E]
   - *If direct disclosure: How did you respond to knowing their sexuality? What were you trying to achieve?*
   - *If indirect (e.g., reading existing formulation): Did you verify this information with the young person? Why? Has the way you engage with them changed after knowing this? How? Why?*
2. Could you describe a memorable moment in your work with them? [E/A]

*Prompt: this can relate to, for example, an ‘a-ha’ moment you had, or working with their family or your colleagues*

- - *What did this experience change in you?*
  - *How have your approaches / knowledge / values / beliefs changed following this experience?*

1. Considering your own experience of working with sexual minority adolescents, what are the key issues that you feel the psychological profession should be talking about and acting on? [R]
   - *How do you think these can be realised?*
2. Is there anything you want to change about your service’s way of working with sexual minority adolescents? [A]
   - *Why? What do you think that could achieve?*
3. In the following question, I’d like you to answer more broadly beyond working with this young person / these young people. From your perspective, how does the wider social context influence how you work with sexual minority adolescents? [R]

*Prompt: this can relate to any historical and/or current societal factors that you are aware of*

1. Is there anything related to working with sexual minority adolescents we haven’t talked about that you want to add?
