# Supplementary material for: Supporting sexual minority adolescents: A critical realist thematic analysis of psychological therapists' experiences
Source: Psychol Psychother. 2026 Mar 5;99(2):684–704. doi: 10.1111/papt.70051 (PMC13162195; doi:10.1111/papt.70051)
Supplement: Supplementary file 2 — Data S2 [file PAPT-99-684-s001.docx]

**Dispositional, Inferential, Experiential Themes, and Counts of References**

| **Dispositional themes (3)** | **Inferential themes (11)** | **Illustrative experiential themes (selected from 161)** | **Total** | **Alexis*** | **David** | **Jocelyn*** | **Moira** | **Ron*** | **Stevie*** | **Twyla*** |
| --- | --- | --- | --- | --- | --- | --- | --- | --- | --- | --- |
| **Psychological therapists’ experience of the socio-environmental forces shaping adolescent sexuality** | *Emerging sexual identity amid developmental complexity*  *(12 experiential themes)* | Adolescence involves exploring identities | **10** |  |  | 4 | 1 | 1 | 3 | 1 |
|  |  | Older adolescents may or may not be more accepting of their sexuality | **4** |  | 1 | 3 |  |  |  |  |
|  |  | Sexuality development can involve confusion | **8** | 1 | 3 | 2 |  | 2 |  |  |
|  |  | Sexuality is one part of a whole | **7** |  | 2 | 1 | 1 | 1 | 1 | 1 |
|  |  | Sexuality still emerging for early adolescents | **10** |  | 1 | 3 |  | 2 | 3 | 1 |
|  | *Navigating stigma and social rejection*  *(13 experiential themes)* | Awareness of SMA’s sensitivity to rejection | **7** |  |  | 1 | 1 |  |  | 5 |
|  |  | Changing societal acceptance may help some SMA to normalise sexuality | **6** |  |  | 3 |  | 1 | 2 |  |
|  |  | Impacts of SMA’s immediate social contexts | **13** |  | 1 | 4 | 1 | 2 | 3 | 2 |
|  |  | Disclosure can lead to negative outcomes | **2** |  |  |  | 1 | 1 |  |  |
|  |  | Shame and internalised homophobia | **12** | 1 | 3 | 3 | 2 | 1 | 2 |  |
|  |  | Some cultures or communities may be less accepting of sexual diversity | **6** |  | 1 |  |  | 1 | 4 |  |
|  | *Adverse experiences shaping but not defining sexual identity (5 experiential themes)* | Some SMA experience social isolation | **2** | 1 |  |  |  |  | 1 |  |
|  |  | Some SMA experienced bullying | **5** | 1 |  |  |  |  | 3 | 1 |
|  |  | Traumatic experiences complicate sexuality development | **7** | 1 |  | 2 | 2 |  |  | 2 |
|  | *Fluidity and tensions in identity labels and self-definition*  *(17 experiential themes)* | Fluidity of sexuality doesn't match categories | **5** |  | 1 | 1 |  | 1 | 2 |  |
|  |  | Peer pressure to conform to sexuality labels | **4** |  |  | 3 | 1 |  |  |  |
|  |  | Labels not always helpful for SMA to explain their experience | **11** |  | 2 | 3 |  | 4 | 2 |  |
|  |  | Labels can provide a sense of belonging to SMA | **2** | 1 |  | 1 |  |  |  |  |
|  |  | Language changes over time | **3** | 2 |  |  |  | 1 |  |  |
|  |  | Others make assumptions on categories that do not fit SMA's experience | **10** |  |  | 2 | 3 | 3 | 2 |  |
|  |  | SMA may or may not have the language or label to identity with | **5** | 1 |  |  | 2 |  | 1 | 1 |
|  |  | Some SMA accept and sure about their sexuality | **9** | 1 |  | 4 |  | 2 | 2 |  |

*Note. Asterisk (*) denotes participants who reviewed and agreed with experiential themes generated from their interview transcript.*

| **Dispositional themes (3)** | **Inferential themes (11)** | **Illustrative experiential themes (selected from 161)** | **Total** | **Alexis*** | **David** | **Jocelyn*** | **Moira** | **Ron*** | **Stevie*** | **Twyla*** |
| --- | --- | --- | --- | --- | --- | --- | --- | --- | --- | --- |
| **Psychological therapists strive to offer attuned, responsive therapy** | *Therapists’ reflective inner dialogue*  *(13 experiential themes)* | 'Park' therapists' own reactions and thoughts | **14** | 2 | 2 | 4 | 4 | 1 |  | 1 |
|  |  | Don't impose therapist's own agenda on SMA | **15** | 1 | 5 | 5 | 2 | 1 | 1 |  |
|  |  | End of therapy may or may not be self-acceptance | **7** |  | 1 |  | 1 | 3 | 1 | 1 |
|  |  | Therapists experience disappointment at ‘incomplete’ ending | **4** |  |  |  |  | 2 |  | 2 |
|  |  | Therapists' pull to rescue and reassure | **6** |  |  | 4 | 2 |  |  |  |
|  |  | Use of supervision to navigate complexity | **3** |  |  |  | 2 | 1 |  |  |
|  | *Empowering adolescents in therapy (10 experiential themes)* | Block out 'noise' - be with SMA in the room | **4** |  | 2 | 1 | 1 |  |  |  |
|  |  | Don't jump in or be leading - stay with the feelings | **8** |  | 1 | 3 | 2 | 2 |  |  |
|  |  | Keep SMA in the 'driver seat' of the therapeutic process | **14** | 1 | 1 | 3 | 2 | 4 | 2 | 1 |
|  |  | Language use dependent on SMA’s choice | **7** | 1 | 3 | 2 |  | 1 |  |  |
|  |  | Sexuality may not be relevant or prioritised | **8** |  |  | 3 | 1 | 1 | 3 |  |
|  |  | Therapists' positive emotions associated with SMA sharing their sexuality | **3** |  | 1 | 1 |  |  |  | 1 |
|  | *Facilitating conversations about sexuality (41 experiential themes)* | Disclosing or outwardly presenting sexuality can be a big thing for SMA | **3** |  |  |  |  | 1 |  | 2 |
|  |  | Don't make disclosure a big deal to normalise sexual diversity | **10** | 1 |  | 4 | 2 |  | 2 | 1 |
|  |  | Exploratory support for SMA may take longer time | **4** | 1 |  | 2 |  |  |  | 1 |
|  |  | Exploring sexuality cannot be rushed | **7** |  | 2 | 1 |  | 1 |  | 2 |
|  |  | Important to protect confidentiality | **6** |  | 1 | 2 |  | 3 |  |  |
|  |  | Learning about SMA’s sexuality indirectly | **3** |  | 1 |  |  | 1 |  | 1 |
|  |  | Matching YP's identity development process | **4** |  | 2 |  |  | 2 |  |  |
|  |  | Open question about relationships helps SMA to feel safe to disclose | **6** |  |  | 1 |  | 2 | 2 | 1 |
|  |  | Relational approaches are more helpful than directive approaches | **4** |  | 2 |  | 1 |  |  | 1 |
|  |  | Supporting self-rejecting SMA to explore sexuality | **5** | 1 | 1 |  |  | 1 | 2 |  |
|  |  | Therapists need to gently 'dig' and create space to talk about sexuality | **6** |  | 1 | 2 |  | 3 |  |  |
|  |  | Therapists prioritise establishing safety and alliance | **3** |  |  |  |  |  |  | 3 |
|  |  | SMA assess safety before sharing their sexuality | **7** |  |  |  |  | 2 |  | 5 |
|  |  | SMA may not share about their sexuality straight away | **4** |  | 1 | 2 | 1 |  |  |  |
|  |  | Therapists' role to guide self-discovery | **7** |  | 2 | 2 | 1 | 1 |  | 1 |
|  |  | Using acceptance to support exploration | **9** |  | 1 | 2 |  | 1 | 1 | 4 |
|  |  | Using curiosity to explore experience (show interest) | **27** | 4 | 2 | 7 | 7 | 3 | 2 | 2 |
|  |  | Using therapeutic relationship to mitigates shame | **4** |  | 2 |  |  |  |  | 2 |
|  | *Commitment to ongoing learning and humility  (20 experiential themes)* | Learning from SMA's lived experience | **8** | 1 |  | 2 | 1 |  | 1 | 3 |
|  |  | Learning from past experiences of supporting SMA | **5** | 1 | 1 | 1 |  | 1 | 1 |  |
|  |  | Learning from research evidence - value + limits | **8** |  | 6 |  | 1 |  |  | 1 |
|  |  | Supporting SMA involves openness to learn and flexibility | **10** | 2 |  | 3 |  | 2 | 3 |  |
|  |  | Therapists' own lived experience can be helpful and unhelpful | **3** |  | 3 |  |  |  |  |  |
|  |  | Therapists' own sexuality development may impact therapeutic work | **2** |  | 2 |  |  |  |  |  |
|  |  | Generalised approach may not fit for specific SMA | **5** |  | 2 |  | 2 | 1 |  |  |
|  |  | Worry about biased research or information | **2** | 1 |  | 1 |  |  |  |  |
|  | *Systemic collaboration beyond individual therapy*  *(6 experiential themes)* | Limits of direct therapeutic work | **2** |  |  |  |  | 1 |  | 1 |
|  |  | More joint-up support between services can be helpful | **3** |  | 2 |  |  |  |  | 1 |
|  |  | Professional to think together on how to support SMA | **3** |  |  | 2 |  |  |  | 1 |
|  |  | Support both SMA and family to understand SMA | **1** |  |  |  |  | 1 |  |  |
| **Wider socio-political tensions influence how therapists navigate identity-related work** | *Navigating professional polarisation and discourse tensions  (18 experiential themes)* | Avoid black & white thinking amongst professionals | **3** |  |  | 1 | 1 |  | 1 |  |
|  |  | Diverse views not welcomed within the profession | **6** | 1 |  | 4 | 1 |  |  |  |
|  |  | Perceived judgement from others create fear within the profession | **10** | 4 |  | 4 | 2 |  |  |  |
|  |  | Social media amplifies fear for 'saying the wrong thing' | **1** | 1 |  |  |  |  |  |  |
|  |  | Therapists need to be aware of socio-political context | **7** |  | 4 |  | 2 |  |  | 1 |
|  |  | Worry of being in an echo chamber | **3** | 1 |  | 1 |  |  |  | 1 |
|  |  | Non-judgemental professional conversations needed | **5** | 1 |  | 3 | 1 |  |  |  |
|  |  | Safety within and across professions is lacking | **3** | 1 |  | 2 |  |  |  |  |
|  | *Complexities and concerns around gender identity and SMA wellbeing  (6 experiential themes)* | Concerns about gender issues negatively impact SMA | **5** | 2 |  |  | 3 |  |  |  |
|  |  | Different degree of desire to work with gender vs sexuality issues | **3** |  |  | 1 |  |  | 2 |  |
|  |  | Gender can get muddled with sexuality issues | **6** | 1 |  | 4 | 1 |  |  |  |
|  |  |  |  |  |  |  |  |  |  |  |
